# Characterization and valorization of soybean residue (okara) for the development of synbiotic ice cream

**DOI:** 10.1002/fsn3.3606

**Published:** 2023-08-07

**Authors:** Rimsha Farooq, Fedrick C. Mgomi, Farhan Saeed, Aftab Ahmad, Aasma Asghar, Sakhawat Riaz, Huda Ateeq, Yasir Abbas Shah, Mahbubur Rahman Khan, Yi Li, Muhammad Afzaal

**Affiliations:** ^1^ School of Food Science and Engineering Yangzhou University Yangzhou Jiangsu Province China; ^2^ Food Safety and Biotechnology Laboratory, Department of Food Science Government College University Faisalabad Pakistan; ^3^ Department of Nutritional Sciences Government College University Faisalabad Pakistan; ^4^ Department of Home Economics Government College University Faisalabad Pakistan; ^5^ Department of Food Processing and Preservation Hajee Mohammad Danesh Science & Technology University Dinajpur Bangladesh

**Keywords:** agro‐waste, functional food, prebiotic, probiotics, soybean, synbiotic ice cream

## Abstract

There is an increasing challenge in probiotic viability and stability during food product formulation, processing, and storage. However, synbiotic functional foods have promising potential to deliver the targeted benefits. This study aimed to isolate the okara from soybean residue, and obtained okara flour was further characterized using Fourier transform infrared spectroscopy (FTIR) and scanning electron microscope (SEM). Synbiotic ice cream was developed by fortification with *Lactobacillus rhamnosus* GG and okara at different concentrations (1–3%). Additionally, the synbiotic ice cream was subjected to physicochemical and sensory attributes over 60 days of storage. High viability of *L. rhamnosus* GG (8.17 log CFU/mL) was observed during storage at 3% okara. Moreover, adding okara at 2% or higher improved viscosity, reduced overrun, and maintained probiotic viability. When compared to the control (ice cream without okara), synbiotic ice cream exhibited a higher protein content and a lower fat level. The synergistic combination of probiotics and okara in ice cream is a potentially novel approach for developing functional ice cream. The addition of okara is not only helpful in increasing the nutritional value of the ice cream but will also be a way forward to minimize agricultural waste. Synbiotic ice cream developed in this study may be considered a potential functional food rich in protein and low in fat.

## Graphical abstract

The synergistic combination of probiotics and soybean residue (okara) in ice cream is a potentially novel approach for developing functional ice cream. Adding okara (soybean residue) helps increase the nutritional value of the ice cream as well as a way to minimize agricultural waste.

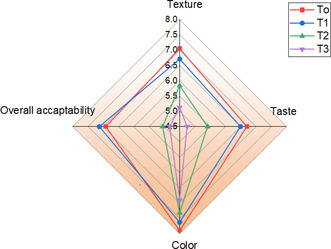

## 1 INTRODUCTION

For years, an increasing demand for health‐boosting food has grown globally. To date, the demand for functional dairy products such as ice cream is getting more attention than other dairy products. Ice cream is a popular frozen dairy product with a satisfying flavor and texture that is mostly liked by all ages (Syed et al., 2018). In addition, ice cream is one of the best carriers for delivering probiotic bacteria (Zoumpopoulou et al., 2021). Various food ingredients, including milk, eggs, colors, sweeteners, emulsifiers, stabilizers, and flavors, have been used to make ice cream (Jain & Rai, 2018). In recent years, due to the high demand for food products promoting good health, the popularity of ice cream usage and its accessibility led to the creation of functional ice cream that contains probiotic bacteria and prebiotics (Ayar et al., 2018). Soybean processing industries produce a huge quantity of agro‐waste known as “okara.” Okara is a highly nutritious waste having a significant amount of dietary fiber, protein, and lipids with promising potential for its application in various food industries (Suzuki & Banna, 2021).

Moreover, due to its rich nutritional contents that include 50% dietary fiber, 25% protein, 10% lipid, and other content (15%) like vitamins, and trace elements, okara is regarded as a cheap source of carbohydrates and proteins and may be used as a prebiotic (Colletti et al., 2020; Muliterno et al., 2017). Prebiotics are considered non‐digestible dietary components that help to provide health benefits to the host by selectively promoting the action or growth of one or a small number of health‐promoting bacteria in the bowel (Bandyopadhyay & Mandal, 2014; Nooshkam et al., 2018). Okara is frequently tossed into landfills or used as feed and fertilizer for livestock. In addition, okara in various food products not only improves the nutritional properties of the product but also has an impact on increasing the probiotic potential (Zinia et al., 2019).

On the other hand, probiotics are non‐pathogenic bacteria that improve human health and help to prevent various diseases, including cancer and cardiovascular disease, when consumed in adequate amounts (Niamah et al., 2017; Song et al., 2015). Probiotic bacteria, *Lactobacillus rhamnosus* GG, is a thoroughly researched bacterial strain with well‐established probiotic characteristics and have been widely employed in the food sector, particularly in yogurt, cheese, ice cream, and drinks (Gao et al., 2021). It is well known to colonize in the gut and is effective against microorganisms that cause rotavirus infection in children and diarrhea in travelers (Mantegazza et al., 2018). The *L. rhamnosus* GG strain may establish a large colony and adhere to the gut lining because it is resistant to bile and acid, making it difficult to eradicate (Salminen et al., 2002).

Synbiotic dairy products, like synbiotic ice cream, are valuable sources of prebiotics which may help increase the number of probiotics and enhance the probiotic potential in the human body with numerous health‐promoting effects (El‐sayed et al., 2014). The consumption of food products, known as “synbiotics,” is highly suggested for the young and old and for particular populations to promote health. Infant growth was reportedly boosted using synbiotic ingredients in the newborn formula (Mazzola et al., 2015). The main difficulty in ice cream production includes storage time, which greatly impacts the viability of probiotics, taste, shelf life, and fat content (Mohammadi et al., 2011).

Considering the prebiotic properties of okara, the current study was planned to evaluate the functional exploration of okara‐supplemented synbiotic ice cream. Furthermore, the physicochemical and sensory properties of okara were evaluated along with probiotic potential during the frozen storage of 60 days.

## 2 MATERIALS AND METHODS

### 2.1 Isolation of okara

Soybean was collected from Ayub Agricultural Research Institute Faisalabad, Pakistan. The okara was extracted according to the Japanese technique as previously described by Swallah et al. (2021). In brief, the soybeans were soaked overnight, boiled at 98°C for 5 min, and then pulverized and filtered. The filtered solid residues were the resulting product, which is called okara. The okara samples were dried in the oven (MemmertGmBH, German) for 24 h at 40°C. The dried samples were then crushed into a fine powder using a micro sieve shaker to get consistent particle size. Prior to further investigation, the samples were kept in sealed bags.

### 2.2 Proximate analysis of dried okara

The Association of Official Analytical Chemists AOAC (2005) technique was used to estimate the total moisture, ash, fat, protein, and crude fiber of the dried okara sample. A 2 g of okara powder was put in a pre‐weighed container and kept in a hot air oven at 105°C. When the constant weight was achieved, total moisture content was determined by weighing the sample again and comparing it to its original weight (on a dry basis). The amount of ash was determined by incinerating a 2 g sample in a crucible and then placed in a muffle furnace at 550°C for 5 h. The total fat content was measured by extracting a 2 g sample in petroleum ether for 5 h and determined using Soxhlet methodology. The protein content of okara samples was measured using the Kjeldahl apparatus. In the Kjeldahl digestion flask, 2 g of dried okara sample was digested. The digested material was added along with 10 mL of diluted sample and 40% NaOH for distillation. During this process, ammonia gas was collected in a solution containing 2% boric acid, a methyl red indicator. Afterward, the solution was distilled by adding 0.1 mL HCl until light pink appeared. Nitrogen percentage was calculated based on the following equation:

$$N\%=\frac{\text{Volume of the}\;0.1\;N\;\mathrm{HCL}\times 0.0014\times \text{Dilution volume}}{\text{Sample weight}\times \text{Volume of the distillate sample taken}\;}\times 100$$

Following the enzymatic method adopted by Ambawat and Khetarpaul (2018), dietary fiber contents were measured, estimating the sum of the soluble and insoluble contents. For insoluble dietary fibers, extraction of water‐soluble components was done using 2 g of sample for 40 min at 60°C and pH was adjusted from 6.0 to 6.5. The suspension was then cooled to 20°C, incubated overnight, and filtered. To get insoluble dietary fiber, the residue was cleaned with acetone, alcohol, and water before being dried for a whole night at 70°C in a vacuum oven. To determine the soluble dietary fibers, the filtrate was acidified with HCl maintaining the pH to 2 to facilitate the precipitation of polysaccharides. Ethanol of four volumes was added slowly and waited for 1 h. Filtered the precipitate, washed with 75% ethanol, and dried at 70°C for 12 h. The residue was weighed in the crucible to give the soluble dietary fiber content of the original material.

### 2.3 Morphological and molecular characterization of isolated okara

#### 2.3.1 Scanning electron microscopy (SEM)

The micrographs of the dried okara were obtained using SEM (cube series emcraft South Korea) as previously described by dos Santos et al. (2019). The okara sample was gold‐plated by ion sputtering, and the micrographs were performed at 10 kV accelerating voltage at 1192 and 6606 times.

#### 2.3.2 Fourier transform infrared spectroscopy (FTIR)

The FTIR spectra of okara were done following the process described by Quintana et al. (2017). The spectra were measured on KBr pellets made in a 1:200 okara–KBr ratio, in the 4000–500 cm^−1^ range. Co‐adding 64 images with a spectral resolution of 4 cm^−1^ allowed for their transmission mode recording.

### 2.4 Preparation of probiotic blend of okara

1% of the *L. rhamnosus* GG inoculum was prepared in the Man Rogosa and Sharpe (MRS) broth and incubation at 37°C overnight. The bacterial cells were then centrifuged and washed twice in sterile peptone water. 100 mL of sterilized full cream milk was combined with 1 mL of *L. rhamnosus* GG (6.5–7.5 log10 CFU/mL) after 25 h of incubation. The resulting mixture was mixed with different concentrations of okara 1%, 2%, and 3% while continually stirring. The probiotic blend of okara was then used to formulate functional synbiotic ice cream.

### 2.5 Formulation of functional synbiotic ice cream

The ice cream base was formulated as per the procedure illustrated by Ibrahim et al. (2022) with slight modifications using ingredients that include milk fat (6%), sugar (16%), milk solids without fat (12%), and stabilizer (0.5%). Briefly, the formulation was initiated with the dispersion process of milk (370 g), powdered milk (55 g), and sugar (90 g) with continuous stirring for 10 min. To produce a creamy emulsion, egg yolk (40 g) was added to bind the fat and water, and the mixture was heated at 78°C for 25 min while constantly stirring. The temperature of the ice cream was maintained while cooking using the double boil method. The mixture was cooled at 40°C, and the ice cream base was then augmented with a probiotic blend of okara at different concentrations: 1% (*T*_1_), 2% (*T*_2_), and 3% (*T*_3_). The control sample was prepared by mixing 1 mL of *L. rhamnosus* GG without okara (*T*_0_). Before physicochemical analysis was carried out, all of the samples were placed into plastic containers with a volume of 250 mL. The mixture was then aged for 20 h at 4°C. For physicochemical, microbiological, and sensory investigation, all samples were stored in a refrigerator at −20°C.

### 2.6 Physicochemical analysis of synbiotic ice cream

#### 2.6.1 Evaluation of fat, protein, and total dietary fiber

Fat, ash, total dietary fibers, and protein were evaluated by AOAC (2005) method.

#### 2.6.2 Determination of overrun

Based on the method stated by Rinaldi et al. (2014), the overrun of the sample was determined. A specified volume of the mixture and ice cream samples contained *L. rhamnosus* GG without okara (*T*_0_), *L. rhamnosus* GG + 1% okara (*T*_1_), *L. rhamnosus* GG + 2% okara (*T*_2_), and *L. rhamnosus* GG + 3% okara (*T*_3_) were weighed and calculated as shown below:

$$\text{Overrun}\%=\frac{\mathrm{IA}-\mathrm{IB}}{\mathrm{IB}}\times 100$$

Whereby: IA and IB represent the corresponding weights of the ice cream mixture and the actual ice cream, respectively.

#### 2.6.3 Melting properties

The melting behavior of ice cream was performed by the method described by Elkot et al. (2022). 100 g of each treatment sample *T*_0_, *T*_1_, *T*_2_, and *T*_3_ were placed on a steel mesh screen and allowed to melt for 90 min at room temperature. The melting rate was then calculated according to the following equation:

$$\text{Melting rate}\;\left(\%\right)=\frac{\text{Weight of melted}\;\mathrm{ice}\;\text{cream}\;\left(g\right)}{\text{Initial weight of}\;\mathrm{ice}\;\text{cream}\;\left(g\right)}\times 100$$

#### 2.6.4 Measurement of total soluble solid (TSS)

According to the method of Halim et al. (2014), a refractometer with a degree Brix (°Brix) specification was used to determine the total soluble solids of the ice cream treated with okara *T*_0_, *T*_1_, *T*_2_, and *T*_3_ at room temperature.

#### 2.6.5 Ice cream viscosity

By following the method described by Baú et al. (2014) and using a viscometer (Bohlin Model Visco 88; Bohlin Instruments), the viscosity of the chilled ice cream (*T*_0_, *T*_1_, *T*_2_, and *T*_3_) was determined at 25 ± 1°C.

#### 2.6.6 Hardness analysis

A texture profile analyzer was used to determine the hardness of ice cream (*T*_0_, *T*_1_, *T*_2_, and *T*_3_) as described by Acu et al. (2020).

#### 2.6.7 pH determination

The pH of the ice cream samples *T*_0_, *T*_1_, *T*_2_, and *T*_3_ were measured using a digital pH meter. Measurements were done in three replicate samples, and results were written as mean ± SD.

### 2.7 Enumeration of probiotic bacteria

Ice cream samples were evaluated for probiotic enumeration with predefined intervals on the 1, 20, 40, and 60 days of storage. Probiotic enumeration was carried out, as previously reported by Nematollahi et al. (2016). Briefly, 1 g of each sample *T*_0_, *T*_1_, *T*_2_, and *T*_3_ were added in 9 mL of peptone water. The mixture was then homogenized for 1 min using a vortex machine. The sample was serially diluted before being counted. After that, the plate was incubated anaerobically for 48 h at 37°C. Probiotic bacteria total plate counts were represented as log10 CFU/mL.

### 2.8 Sensory evaluation

The sensory analysis rating procedure of synbiotic ice cream was employed following the protocol proposed by Falah et al. (2021). At the food sensory laboratory, GCUF Faisalabad, a blind panel of 40 people (20 males and 20 females, all between the ages of 22 and 28) participated in a randomized trial to evaluate the sensory attributes of functional synbiotic ice cream formulated with varying percentages of okara (*T*_0_, *T*_1_, *T*_2_, and *T*_3_). The panelists received 15 min training sessions prior to the examination to become comfortable with the attributes and scaling methods. Color, taste, texture, and general acceptability were the sensory features that the panelists were asked about and were scored on a scale of 1–8, whereby 1–2 represent “don't like at all,” 3–4 represent “slightly dislike,” 5–6 represent “like,” and 7–8 represent “extremely like.”

### 2.9 Statistical analysis

The two‐way analysis of variance (ANOVA) was used to compare the results of the physicochemical, sensory, and microbiological analysis for statistically significant at 95% (*p* < .05). Various okara contents (1–3%) were considered as independent variables, whereas physicochemical, microbiological, and sensory analysis data were placed as dependent variables. The analysis was conducted using the 64‐bit edition of IBM SPSS statistics. However, the statistics were reported as mean and standard deviation (SD).

## 3 RESULTS AND DISCUSSION

### 3.1 Composition analysis of okara

According to the proximate composition results, the okara sample contained an average amount of 13.66% protein, 7.14% fat, 4.44% ash, 33.34% insoluble dietary fiber, and 1.19% soluble dietary fiber, as shown in Table 1. The total dietary fiber found in the current study was relatively lower (34.53%) than reported by Ibrahim et al. (2022) (36.85%). Another study conducted by Mateos‐Aparicio et al. (2010) reported that dried okara contained a high level of total dietary fiber (55%), predominantly insoluble (50%), low‐soluble (5%), and protein (30%). It has been reported that dietary fibers are crucial for a variety of biological processes as well as the prevention of syndromes with various causes (Kamble & Rani, 2020). Okara can be considered an inexpensive fiber‐rich product that can be supplemented into different types of food and exert its beneficial effect.

**TABLE 1:** Mean values for proximate analysis of okara.

Components	Composition (%)
Moisture	3.42 ± 0.05^f^
Protein	13.66 ± 0.16^c^
Fat	7.14 ± 0.03^d^
Ash	4.44 ± 0.01^e^
Insoluble dietary fiber	33.34 ± 0.84^b^
Soluble dietary fiber	1.19 ± 0.01^g^
Total dietary fiber	34.53 ± 1.15^a^

*Note*: Values are represented as mean ± SD followed by least significant difference in the superscripts.

### 3.2 Scanning electron microscopy (SEM)

**FIGURE 1:**
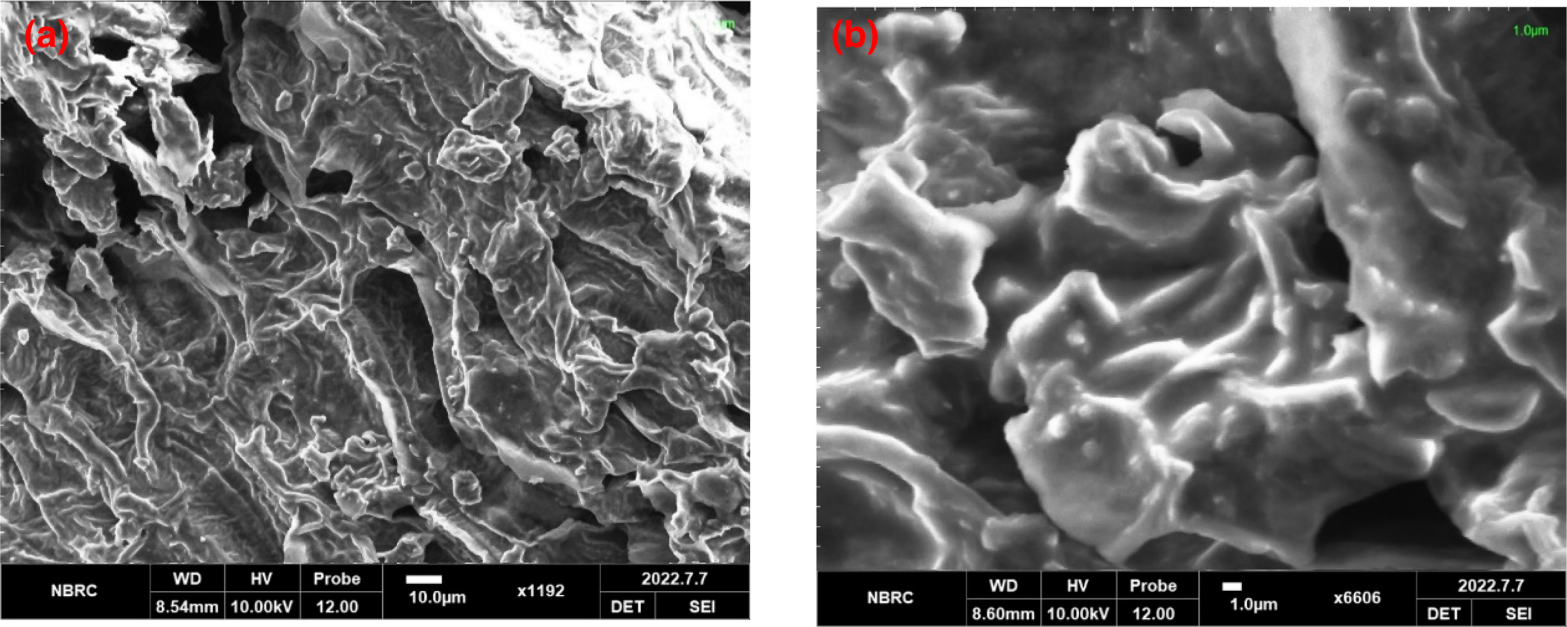
Scanning electron micrographs of okara at magnification of 1192 (a) and 6606 (b).

SEM microscopy images revealed the morphological features of okara, as shown in Figure 1. The figure showed a rough, hollow sphere structure, and the fiber bundle exhibited an ordered structure determined by the particle properties of okara. Unusual forms that were hard to distinguish existed alongside irregular structures. The SEM pictures clearly showed that okara has a rough structure which might be caused due to the presence of cellulose, hemicellulose, and polysaccharides. A study presented by Wu et al. (2020) showed that okara possessed uneven structures made up of large, unusual‐shaped particles divided by big holes. Likewise, the images obtained in the present study showed that okara has a large porous structure.

### 3.3 Fourier transform infrared spectroscopy (FTIR)

**FIGURE 2:**
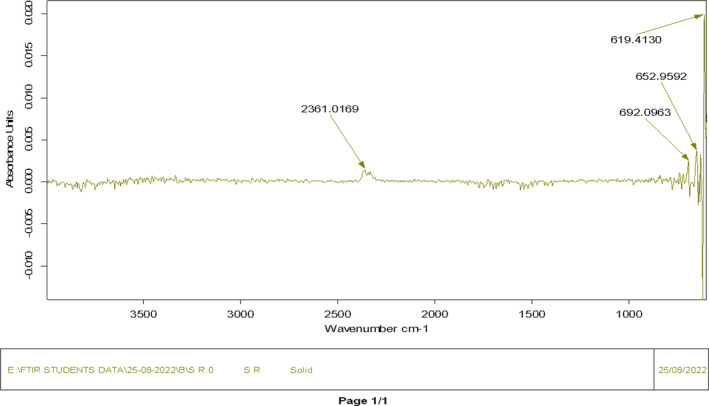
FTIR spectrum of dried okara showing different functional groups and chemical structure.

The FTIR analysis determined the chemical structure and functional groups of okara (Figure 2). The figure showed that the okara exhibited a similar general spectral profile. However, there were some distinctive variations in the peak intensities and spots for several identifying bands. A weak band at 2361 cm^−1^ was detected, which could be ascribed to C‐H group widening in the methylene group of polysaccharides, displaying the presence of typical structure for cellulose and hemicellulose. The small, sharp band at 692, 652, and 619 cm^−1^ are characteristics of β‐glycoside bonds, which are present in hemicellulose. A similar finding was reported by Ullah et al. (2017); they observed the band at 2923 cm^−1^ that showed C‐H group stretching and a peak at 892 cm^−1^ representing β‐glycosidic bonding.

### 3.4 Physicochemical analysis

#### 3.4.1 Composition of synbiotic ice cream

The results of this study demonstrated a decreasing trend in the fat contents with an increase in okara concentration. A high amount of fat (21.78%) was observed in *T*_0_. However, there was a decrease in fat level when okara concentration was increased. Interestingly, in the *T*_2_ treatment, the ice cream had a slightly high‐fat content (16.75%) than in *T*_1_ (14.90%) and *T*_3_ (12.87%). There were no significant differences between the treatment groups (*p* > .05). The decrease in fat content could be due to the high percentage of insoluble fibers in the increased concentration of okara, which potentially promote fat absorption. A study performed by Tong et al. (2014) reported that dietary fibers contain special properties that include the ability to hold oil and water associated with its hydration ability which is supported by the polysaccharide components and chemical structure, and other features like temperature, stress, porosity, solution type of ions, pH, particle size, and ionic strength.

**FIGURE 3:**
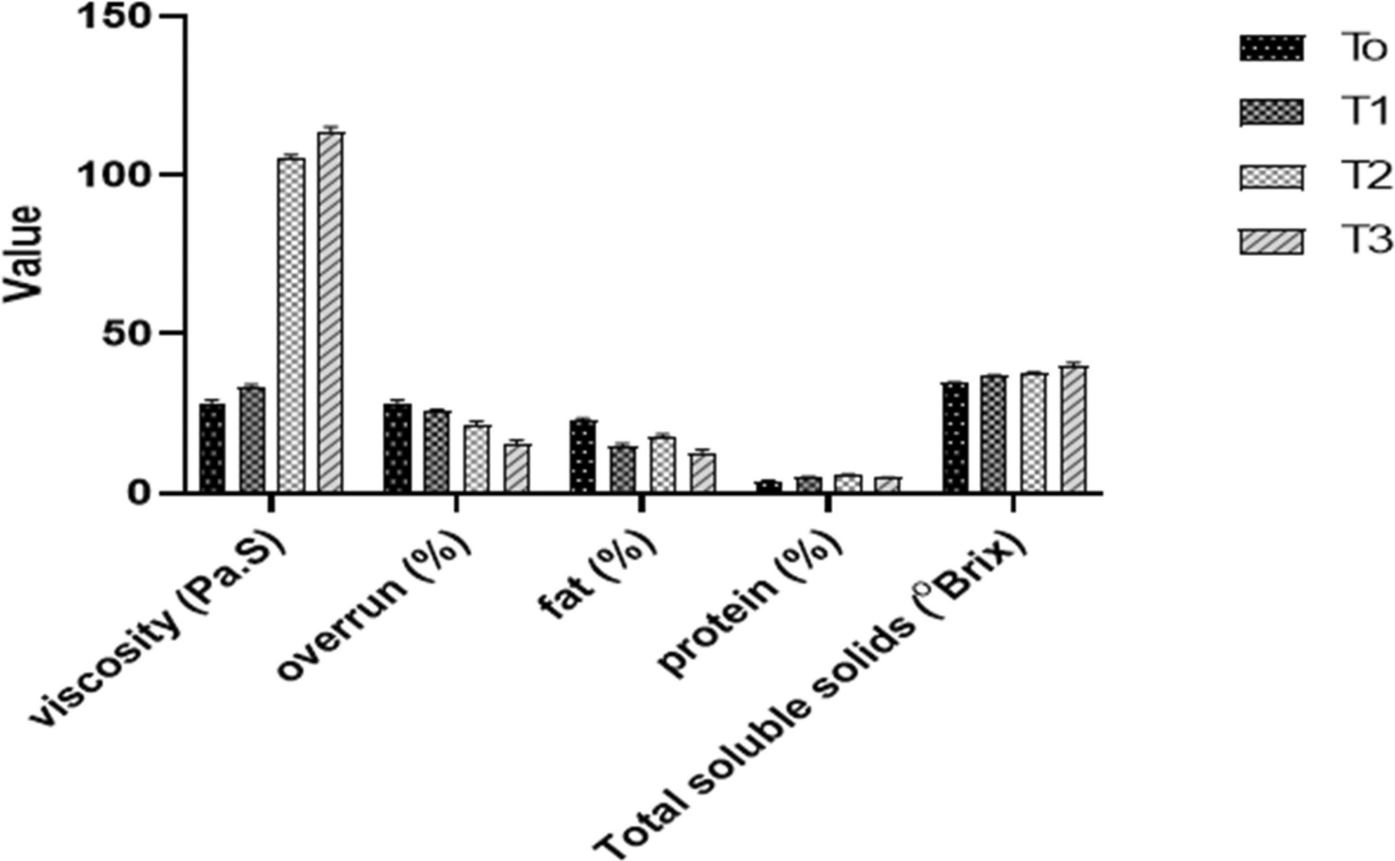
Physicochemical parameters (protein, fat, overrun, viscosity, and total soluble solids) of synbiotic ice cream. *T*_0_ = Control (*Lactobacillus rhamnosus* GG without okara), *T*_1_ = *L. rhamnosus* GG + 1% okara, *T*_2_ = *L. rhamnosus* GG + 2% okara, *T*_3_ = *L. rhamnosus* GG + 3% okara. Mean ± SD.

Moreover, protein contents act as a key functional role in reducing ice recrystallization throughout the static freeze phase, which in turn reduces the formation of ice crystals. This might be due to the ability of proteins to bind to the surface of ice crystals and inhibit their growth, thus helping to maintain the desired texture of the ice cream. In particular, certain types of proteins, such as whey protein and milk protein concentrate, have a high molecular weight and can form a protective layer around the ice crystals, preventing them from growing and agglomerating (Cavender & Kerr, 2020; Mostafavi et al., 2017). In this study, the protein content of synbiotic ice cream was determined using proximate analysis; results are shown in Figure 3. A lower percentage of protein was observed in all samples; however, *T*_2_ had a slightly higher (5.62 ± 0.55%) percentage than other tested samples and the control (3.25 ± 0.63%). Okara is a possible solid fermentation substrate that can decrease or decompose several anti‐nutritional components, including saponin, lectin, and trypsin, to create microbial protein feed (Mok et al., 2019). However, based on our findings, it was seen that different concentrations of okara increased the protein level in the synbiotic ice cream. Indirectly, the enhanced melting rate of functional synbiotic ice cream was caused by a slight increase in protein level compared to low protein content in control, which made it harder. Because synbiotic ice cream developed by adding 2% and 3% okara softened the texture and took longer to melt. Thus, determining protein content helps manufacturers ensure consistent product quality. Knowing the protein content of ice cream helps us make informed decisions about our food choices and ensure that we consume adequate amounts of protein.

#### 3.4.2 Overrun and melting rate

Overrun is the percentage of ice cream expansion that results from the addition of air during the freezing process (Falah et al., 2021). In comparison to the *T*_0_ treatment (28.25%), the overrun for synbiotic ice cream in *T*_1_, *T*_2_, and *T*_3_ ranged from 14.50% to 24.93% (Figure 3). The study demonstrated that the supplementation of various okara levels affected the overrun of the ice cream. The *T*_0_ treatment of ice cream achieved the largest overrun, while *T*_3_ accelerated the lowest overrun. Ice cream overrun was reduced due to the amount of okara because okara is a fiber‐rich food, and hence, decreased the overrun in all treatments. Additionally, a slower melting rate occurs due to the entrapment of air bubbles in the ice cream and can act as an isolator medium caused by a larger overrun (Hanafi et al., 2022). According to research by Salem et al. (2005), probiotic ice cream's shelf life can be increased by reducing overrun.

On the other hand, the *T*_0_ had the maximum melting rate (97.82%), followed by the *T*_1_, *T*_2_, and *T*_3,_ which had 75.54%, 19.93%, and 18.77%, respectively, shown in Table 2. This result demonstrates that the addition of okara decreased the melting rate since the fiber had a high‐fat level. Furthermore, the overrun was also linked to the ice cream melting rate. The results of this experiment showed that the increase in okara concentrations decreased the overrun, which in turn slowed down the melting rate. The recent results confirm those of Wu et al. (2019), who stated that the melting rate increases with overrun. Additionally, the inclusion of partially combined fat from whipped cream, milkfat, and egg yolk created a fat link that may regulate the air pocket and foaming in the ice cream structure, slowing the melting rate of ice cream. Ibrahim et al. (2022) stated that ice cream melts more slowly as okara content rises.

**TABLE 2:** Mean values for melting rate of ice cream.

Treatment	Melting rate% (days)				
	1	20	40	60	Mean
*T*_0_	98.00 ± 0.95^a^	97.84 ± 0.01^b^	97.78 ± 0.02^b^	97.63 ± 0.03^c^	97.82 ± 0.04^a^
*T*_1_	75.69 ± 0.03^d^	75.60 ± 0.02^e^	75.51 ± 0.02^f^	75.39 ± 0.03^g^	75.54 ± 0.20^b^
*T*_2_	20.10 ± 0.03^h^	19.99 ± 0.02^i^	19.90 ± 0.03^j^	19.75 ± 0.30^k^	19.93 ± 0.10^c^
*T*_3_	18.92 ± 0.01^l^	18.84 ± 0.10^m^	18.72 ± 0.02^n^	18.62 ± 0.03°	18.77 ± 0.01^d^
Mean	53.18 ± 0.02^a^	53.06 ± 0.10^b^	52.97 ± 0.20^c^	52.85 ± 0.05^d^	

*Note*: Values are represented as mean ± SD followed by least significant difference in the superscripts.

#### 3.4.3 Viscosity and TSS

The results related to viscosity and TSS are shown in Figure 3. The obtained results exhibited that the incorporation of okara directly affects the viscosity and TSS contents of the ice cream. Ice cream samples in *T*_3_ showed the highest TSS (39.00°brix) followed by *T*_2_ (37.22°brix) and *T*_1_ (36.42°brix). The TSS results for *T*_2_ were slightly greater than *T*_1_, indicating a considerable rise in viscosity level. Contrary to our findings, numerous studies have reported a low TSS value in ice cream with a high water‐freezing potential, contributing to more ice crystals and affecting the texture of the ice cream (Beegum et al., 2021; Pintor et al., 2017). The texture can directly impact the consumer's perception of the ice cream's quality. In addition, the balance between the ingredients of the ice cream is crucial for quality maintained by the control of TSS. All ice cream samples with varying concentrations of okara had significantly varied (*p* < .05) viscosity values as shown in Figure 3. The amount of okara in the mixture directly correlated with a rise in the viscosity level of the ice cream. For instance, the ice cream with the highest viscosity value contained 3% okara. The viscosity was then reduced in the ice cream containing a low concentration of okara and in the control group. This scenario might be caused by the properties of okara fiber in its insoluble and soluble forms, which increased the viscosity of the fiber‐enriched ice cream. The increase showed how fiber has a major impact on the viscosity and smoothness of the ice cream. This conclusion was confirmed by Wang et al. (2020), who proposed that higher viscosity can be attained by combining milk protein and fiber or the combination of insoluble and soluble okara. Moreover, the results for viscosity and TSS were also in line with the findings of Ibrahim et al. (2022); they suggested that adding okara to the ice cream increases the viscosity and TSS.

#### 3.4.4 Hardness of ice cream

**FIGURE 4:**
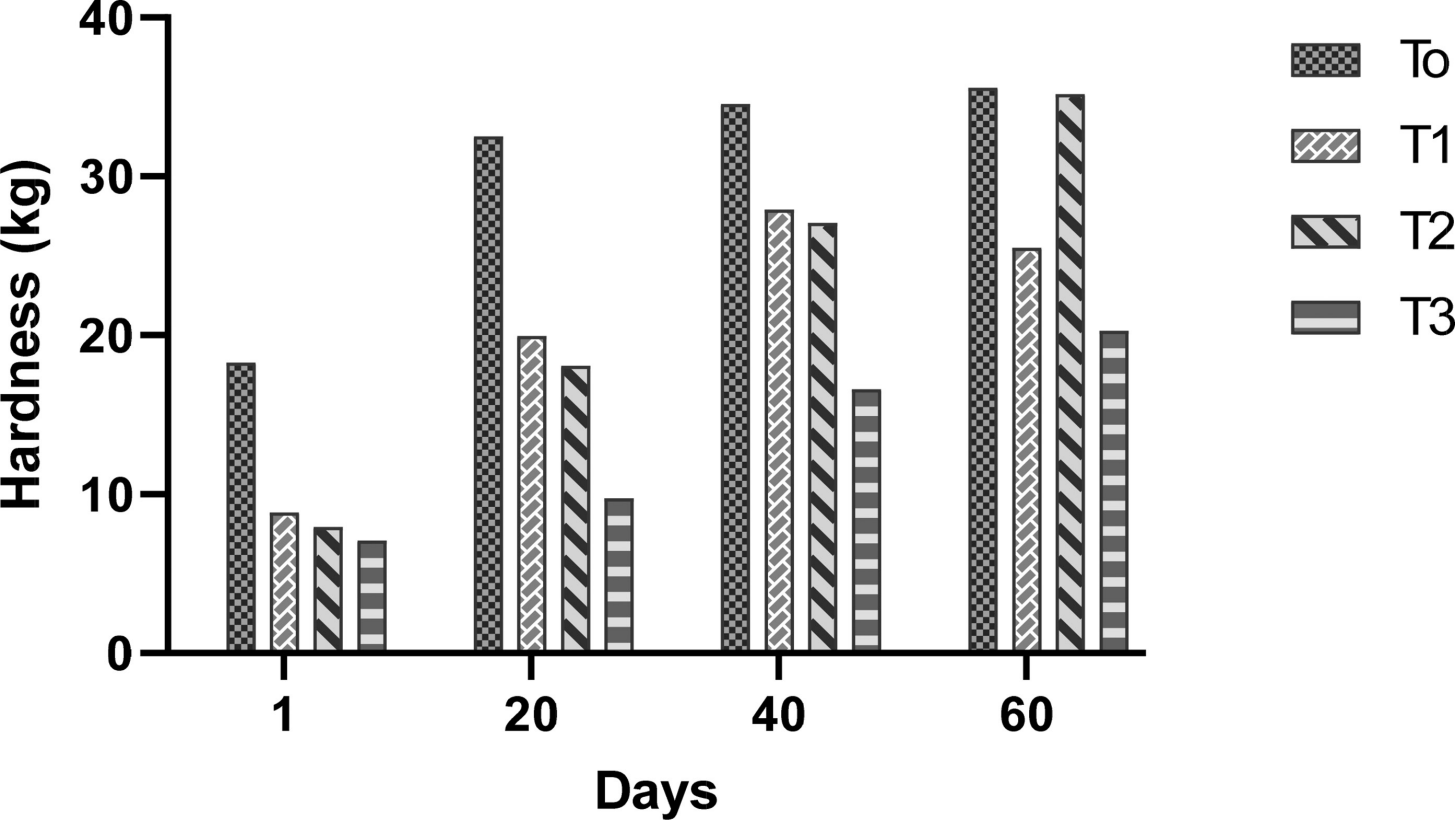
The hardness value of synbiotic ice cream integrated with different concentrations of okara during 60 days of frozen storage. *T*_0_ = Control (*Lactobacillus rhamnosus* GG without okara), *T*_1_ = *L. rhamnosus* GG + 1% okara, *T*_2_ = *L. rhamnosus* GG + 2% okara, *T*_3_ = *L. rhamnosus* GG + 3% okara.

Structural parameter such as hardness is a critical factor in ice cream production because it affects the quality of the ice cream. During storage days 1–60, the incorporation of okara at various concentrations impacted the hardness of the ice cream. Ice cream with various concentrations of okara (1–3%) had significantly (*p* < .05) lower values for its hardness on day 1 than the control group (Figure 4). From days 1–30, the smoothness of the (control) ice cream without okara eventually hardened. The hardness of the ice cream containing okara was not significantly (*p* > .05) different from one another; however, they were significantly (*p* < .05) lower than the control group. On days 40 and 60 of frozen storage, ice creams with okara 1% and 2% had a slight increase in hardness. It is interesting to notice that on the last day of storage (60 days), the ice cream with 3% okara contained no significant impact on the hardness when compared to the control. The differences in hardness during frozen storage might be due to the high fiber contents in okara. In this aspect, adding 3% okara would need little force to penetrate the hardness of the ice cream. In this study, it has been shown that okara contained a high amount of fibers which might be responsible for influencing ice cream texture by increasing the viscosity and freezing point depression by decreasing ice crystals size. This scenario might be influenced by microstructure changes, including fat stability, phase volume, and sizes of ice crystals in the ice cream (Pon et al., 2015). In general, when ice creams are stored in a freezer barrel, ice crystals are formed and continue to expand and harden. It has been shown that two factors are mainly associated with the hardening effect; first, the expansion rate is decreased due to the higher resistance of the mix to water diffusion and the surface of ice crystals; second, increased viscosity promotes crystal melting and attrition (Kumar et al., 2020).

#### 3.4.5 pH value

The results for pH are summarized in Table 3. As the freezing storage period increased to 60 days, the pH level of the ice cream that contained okara became more acidic. pH levels of all ice cream samples on day 1 ranged from 6.95 to 7.03 at a basal level, with no significant (*p* > .05) difference. After 20 days of storage, the pH level in *T*_1_ (6.62) and *T*_2_ (6.64) was higher than in *T*_0_ (6.59) and *T*_3_ (6.61) treatments. There was a slight increase in the pH values on day 40, *T*_0_ (6.53), *T*_1_ (6.64), and *T*_3_ (6.57), while *T*_2_ dropped to 6.55. At day 60, *T*_2_ had a pH level that was significantly (*p* > .05) higher than *T*_1_, *T*_3_, and *T*_0_ group, which was 6.57, 6.50, 6.70, and 6.32, respectively. Our findings demonstrated that *T*_0_ had the lowest pH readings throughout the storage time, ranging from 6.32 to 6.95. This was probably due to the fact that *T*_0_ did not contain okara, thus causing the pH to drop to an acidic level, protecting probiotic bacteria from acidic conditions. Since *L. rhamnosus* GG can tolerate a pH as low as 2–2.5, it is widely considered that probiotic bacteria are more tolerant of acidic circumstances (Reale et al., 2015).

**TABLE 3:** Mean values for pH of ice cream.

Treatment	Days				
	1	20	40	60	Mean
*T*_0_	6.95 ± 0.01^c^	6.59 ± 0.01^g^	6.53 ± 0.20^j^	6.32 ± 0.20^L^	6.59 ± 0.30^d^
*T*_1_	6.95 ± 0.03^c^	6.62 ± 0.02^ef^	6.64 ± 0.10^e^	6.57 ± 0.30^h^	6.69 ± 0.05^b^
*T*_2_	7.00 ± 0.10^b^	6.64 ± 0.02^e^	6.55 ± 0.30^i^	6.70 ± 0.01^d^	6.72 ± 0.10^a^
*T*_3_	7.03 ± 0.20^a^	6.61 ± 0.01^f^	6.57 ± 0.01^hi^	6.50 ± 0.02^k^	6.67 ± 0.20^c^
Mean	6.98 ± 0.03^a^	6.61 ± 0.30^b^	6.57 ± 0.04^c^	6.52 ± 0.10^d^	

*Note*: Values are represented as mean ± SD followed by least significant difference in the superscripts.

#### 3.4.6 Viability of *L. rhamnosus* GG during ice cream storage

**FIGURE 5:**
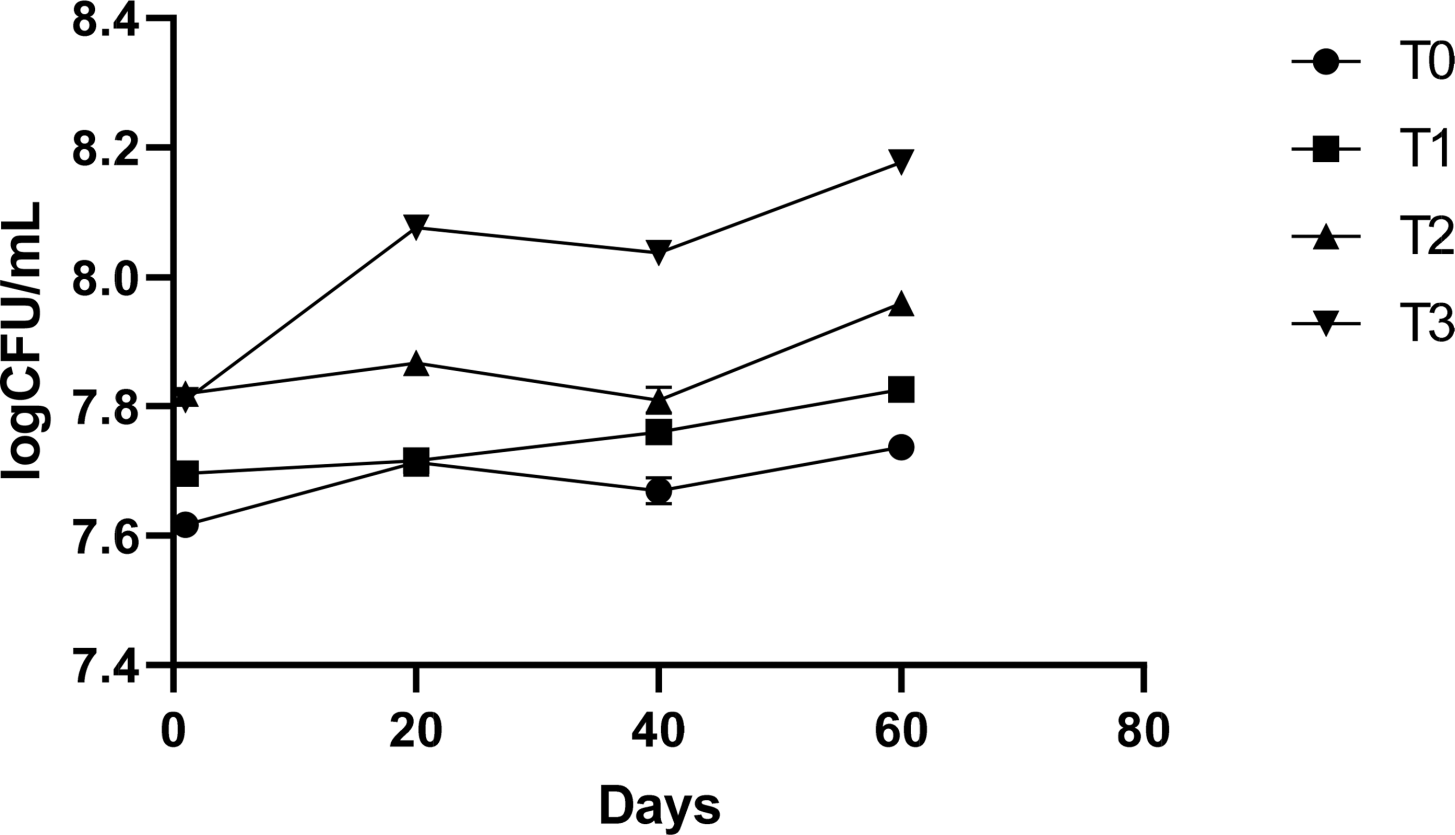
Probiotic cell counts (Mean ± SD) of *Lactobacillus rhamnosus* GG in ice cream supplemented with different concentrations of okara (1–3%) during 60 days of frozen storage. *T*_0_ = Control (*L. rhamnosus* GG without okara), *T*_1_ = *L. rhamnosus* GG + 1% okara, *T*_2_ = *L. rhamnosus* GG + 2% okara, *T*_3_ = *L. rhamnosus* GG + 3% okara.

The probiotic bacteria *L*. *rhamnosus* GG viability was assessed on days 1, 20, 40, and 60; results are shown in Figure 5. The probiotic viability was significantly (*p* > .05) affected during storage. On day 1 no significant difference among the treatment was observed. However, as the storage period increased a rapid change in the viable count was recorded. These differences ranged from 7.60 to 7.82 log10 CFU/mL. On day 20, the viability of *L. rhamnosus* GG in *T*_3_ is greater than in *T*_0_, *T*_1_, and *T*_2_. The viability started to rise in *T*_2_ (7.79 log10 CFU/mL) and *T*_3_ (8.02 log10 CFU/mL). In comparison, *T*_0_ showed the lowest counts of *L. rhamnosus* GG throughout the frozen storage time ranging from 7.60 to 7.82 log10 CFU/mL. Furthermore, the ice cream sample containing okara showed a more stable number of probiotics than the control samples.

In this study, *T*_3_ exhibited the most viable population of probiotic bacteria; this might be due to the highest protein concentration that serves as an essential source of nitrogen for *L. rhamnosus* GG growth. Okara is a significant source for solid‐state fermentation of microbial protein as food since it is thought to be an excellent supply of nutrients, including minerals, lipids, protein, and vitamins. Additionally, dietary fiber present in the okara may serve as a potential substrate for the growth of probiotics and safeguard the probiotics from cold damage and shear stress linked with the integration of small air bubbles during mixing and freezing shock. According to Ibrahim et al. (2022), the presence of oxygen and the impact of hardening during frozen storage both of which are inhibitors of bacterial growth, are other potential causes of the decline in the probiotic count. However, another study conducted by Hanafi et al. (2022) also studied the probiotic enumeration of ice cream using coconut powder as a prebiotic source along with probiotic bacteria. The results suggested an increase in probiotic counts. Another research conducted by Elkot et al. (2022) also prepared synbiotic ice cream using black rice powder as a prebiotic and *L. acidophilus* and determined the probiotic viability during freeze storage. The results of their work indicated an increase in probiotic levels. The incorporation of black rice powder as a prebiotic source improved the viability of *L. acidophilus* in ice cream samples over 60 days of storage that is in line with the findings of the current study.

#### 3.4.7 Sensory acceptability

**FIGURE 6:**
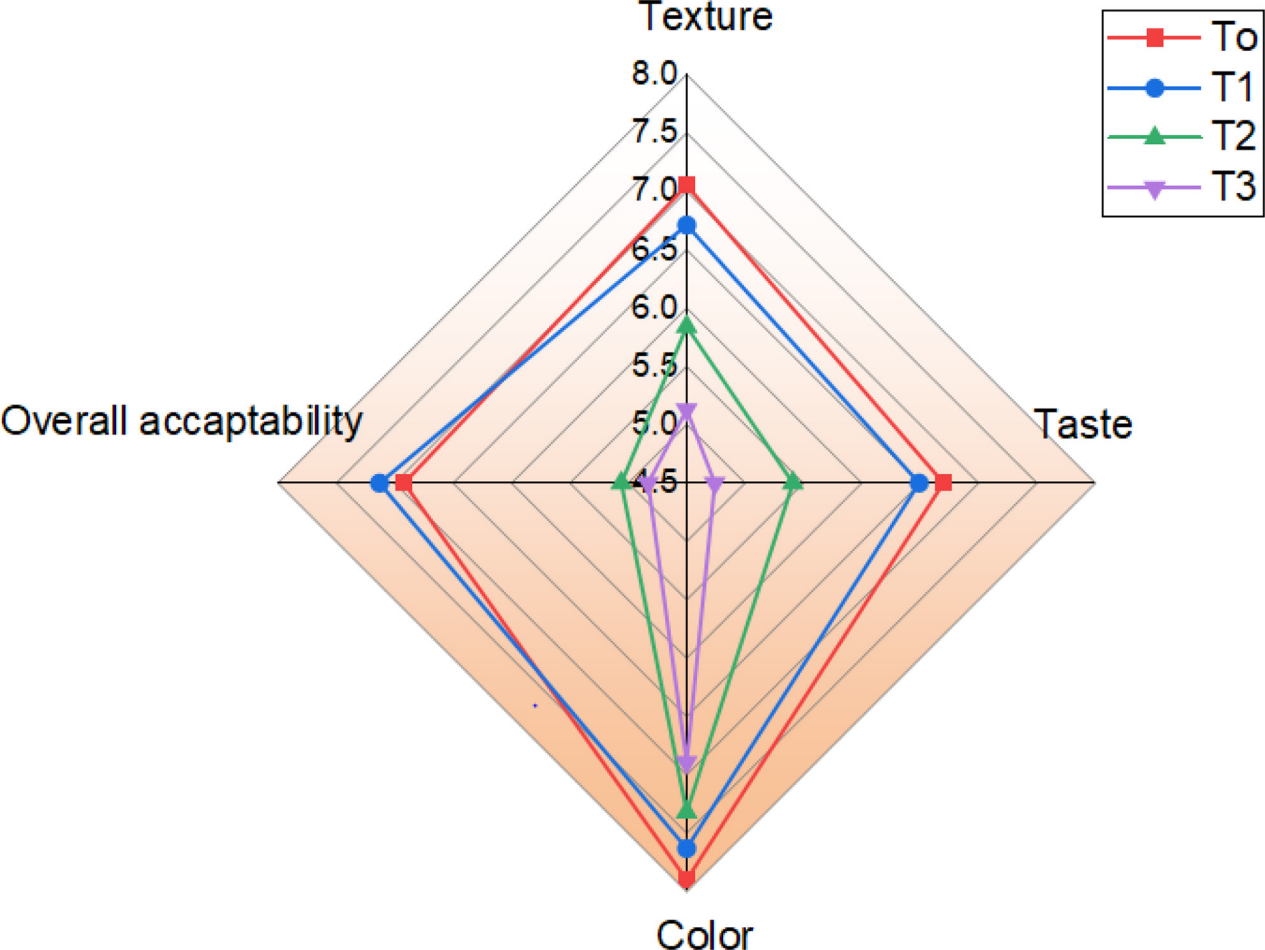
Sensory attributes of synbiotic ice cream are represented graphically (mean ± SD, *n* = 3). *T*_0_ = Control (*Lactobacillus rhamnosus* GG without okara), *T*_1_ = *L. rhamnosus* GG + 1% okara, *T*_2_ = *L. rhamnosus* GG + 2% okara, *T*_3_ = *L. rhamnosus* GG + 3% okara.

Sensory analysis is essential in the ice cream industry to ensure that the final product meets the expectations of the consumers. It involves evaluating the appearance, aroma, texture, and flavor of the ice cream and providing valuable information to producers and manufacturers on how to improve their products. Based on the findings shown in Figure 6, there were no variations in color ratings between the okara‐fortified ice cream and the control that were statistically significant (*p* > .05). Our current study evaluated the sensory analysis of synbiotic ice cream after 60 days of frozen storage (Figure 6). The results showed that *T*_0_ and *T*_1_ had the most favored textures, with mean scores of 6.92 and 6.73, respectively. However, ice creams in *T*_2_ and *T*_3_ had the lowest mean texture scores of 5.84 and 5.13, respectively. The texture of ice cream was seen to be thick due to the addition of more okara. There was a decrease in all tested parameters in *T*_3_, while *T*_1_ showed improved acceptability among others. The ice creams in *T*_0_ and *T*_1_ received the highest ratings from consumers and had considerably higher mean scores because most people assumed that *T*_1_ ice cream sample was tasteless and sandy. However, the sandiness of the *T*_2_ and *T*_3_ okara concentrations was significant, and most people did not like the flavor. This result was well demonstrated by the *T*_3_ ice cream, which received the lowest ratings for appearance, color, flavor, and general acceptance. Undesirable textures like sandiness and a sticky flavor would likely cause reduced overall acceptability. The control sample generally had good acceptability compared to the tested samples. In all tested parameters, color was the most highly scored parameter compared to taste, texture, and overall acceptability. However, there were no significant differences between the control and treated samples.

## 4 CONCLUSIONS

Okara is a rich source of dietary fibers which make it a potential prebiotic. The inclusion of okara in the development of synbiotic ice cream has shown a significant influence on the sensory attributes and physicochemical properties of the ice cream. Furthermore, the addition of okara has also key role in maintaining the therapeutic numbers of probiotic during storage of synbiotic ice cream. The use of okara for the development of functional food is a proactive approach to prevent the community from various health disorders.

## AUTHOR CONTRIBUTIONS

**Rimsha Farooq:** Conceptualization (equal); methodology (equal); writing – original draft (equal). **Fedrick C. Mgomi:** Formal analysis (equal); writing – review and editing (equal). **Farhan Saeed:** Data curation (equal); formal analysis (equal). **Aftab Ahmed:** Data curation (equal); writing – review and editing (equal). **Aasma Asghar:** Validation (equal); writing – review and editing (equal). **Sakhawat Riaz:** Data curation (equal); writing – review and editing (equal). **Huda Ateeq:** Formal analysis (equal); writing – review and editing (equal). **Yasir Abbas Shah:** Data curation (equal); formal analysis (equal); writing – review and editing (equal). **Mahbubur Rahman Khan:** Formal analysis (equal); validation (equal). **Yi Li:** Supervision (equal); writing – review and editing (equal). **Muhammad Afzaal:** Supervision (equal); writing – review and editing (equal).

## FUNDING INFORMATION

This work was supported by the High Level Talents Support Program of Yangzhou University.

## CONFLICT OF INTEREST STATEMENT

The authors declare that they have no conflict of interest.

## ETHICS STATEMENT

This article does not contain any studies with human participants or animals performed by any of the authors.

## INFORMED CONSENT

For this type of study, formal consent is not required.

## CONSENT TO PARTICIPATE

Corresponding and all the co‐authors are willing to participate in this manuscript.

## Data Availability

Even though adequate data has been given in the form of tables and figures, however, all authors declare that if more data is required then the data will be provided on a request basis.
